# Efficacy of Combined Narrow‐Band IPL and Super‐Pulsed CO_2_ Fractional Laser for Early Hypertrophic Scars: Longitudinal Therapeutic Trend

**DOI:** 10.1111/jocd.71079

**Published:** 2026-07-22

**Authors:** Yanan Jiang, Yadan Chen, Demei Zhao, Yu Gao, Yerong Jiang, Qian Tan

**Affiliations:** ^1^ Department of Burns & Plastic Surgery Nanjing Drum Tower Hospital Clinical College of Nanjing Medical University Nanjing Jiangsu China; ^2^ Department of Burns & Plastic Surgery, Nanjing Drum Tower Hospital, Affiliated Hospital of Medical School Nanjing University Nanjing China

**Keywords:** combination therapy, hypertrophic scars, narrow‐band intense pulsed light, super‐pulsed CO_2_ fractional laser

## Abstract

**Background:**

Hypertrophic scars are characterized by excessive microvascular proliferation and abundant collagen deposition. Combined narrow‐band intense pulsed light (NB‐IPL) and super‐pulsed CO_2_ fractional laser (SPCO_2_ FL) have become a promising therapeutic protocol. However, prior studies are limited by inconsistent treatment regimens and insufficient follow‐up.

**Purpose:**

To retrospectively evaluate clinical efficacy of NB‐IPL monotherapy versus NB‐IPL combined with SPCO_2_ FL therapy for early hypertrophic scars at longitudinal follow‐up visits.

**Methods:**

Patients with early hypertrophic scars treated with laser at Nanjing Drum Tower Hospital from February 2023 to May 2025 were enrolled and allocated into the experimental group (combined NB‐IPL and SPCO_2_ FL) and the control group (NB‐IPL monotherapy). All received six treatment sessions at intervals of 8–10 weeks, with evaluations at baseline, mid‐treatment, last treatment, and the 3‐ and 6‐month follow‐ups. Treatment pain, scar appearance, and overall scar status were respectively evaluated using the Visual Analog Scale (VAS), Vancouver Scar Scale (VSS), and Patient and Observer Scar Assessment Scale (POSAS). Pain and pruritus scores received from the POSAS Patient Scale were additionally analyzed. Repeated‐measures ANOVA was adopted for longitudinal data analysis.

**Results:**

Of the 100 initially enrolled patients, 14 were excluded, and 86 patients were finally analyzed (46 combination and 40 monotherapy). POSAS Patient Scale and Observer Scale scores were significantly reduced over time in both groups, with superior improvement observed in the experimental group. Repeated‐measure analysis revealed significant time, group, and interaction effects for pain, while only a significant main time effect existed for pruritus. Both groups obtained significant improvements in pigmentation, vascularization, pliability, and thickness, and the experimental group had better efficacy. No intergroup differences in treatment‐related VAS pain scores were detected, indicating similar treatment pain and tolerability.

**Conclusion:**

Combined NB‐IPL and SPCO_2_ FL yields better improvements in the appearance, pliability, and symptoms of hypertrophic scars than NB‐IPL monotherapy. This combination protocol is an effective strategy for hypertrophic scar treatment.

## 1 Introduction

Although scars are pathologically benign and generally regarded as a trivial clinical problem, they are highly prevalent and affect tens of thousands of people worldwide [1]. As a common type of scar, hypertrophic scars (HTS) arise after deep dermal injury. They are characterized by abnormal vascular proliferation (clinically presenting as erythema) and excessive extracellular collagen deposition (dermal thickening), which are strictly confined to the original wound margin [2]. In the early proliferative phase, HTS appear pink to purplish, occasionally accompanied by hyperpigmentation, pruritus, stabbing pain, and tightness; during maturation, hypervascularity and pigmentation slightly subside, but the hypertrophic appearance generally remains [3]. Accordingly, early intervention to suppress excessive microvessels and collagen synthesis is critical for effective scar treatment.

A variety of traditional techniques have been employed for the HTS treatment, including silicone‐based products, compression therapy, corticosteroids, radiotherapy, cryotherapy, and surgery [4]. Despite their widespread clinical application, these methods show limited efficacy. Their drawbacks include poor patient compliance with silicone‐based products and compression therapy, injection pain and potential endocrine disturbances associated with corticosteroids, oncogenic risks linked to radiotherapy, and high recurrence rates following surgical excision or cryotherapy [5]. Energy‐based devices (EBDs), as non‐invasive modalities, have gradually emerged as core interventions for HTS [6]. As the main category of these devices, narrow‐band intense pulsed light (NB‐IPL) can selectively target and thermocoagulate microvessels, while super‐pulsed carbon dioxide fractional laser (SPCO_2_ FL) induces epidermal ablation to trigger collagen remodeling [7].

Combination therapy using EBDs is widely recognized as an optimal strategy for HTS treatment, and the combined use of NB‐IPL and SPCO_2_ FL has emerged as the most effective treatment protocol [8]. Existing studies on this combination method have been constrained by variable regimens and short follow‐up. Herein, we conducted a retrospective cohort study with six treatment sessions and long‐term follow‐up for HTS. We analyzed the efficacy of combined therapy versus NB‐IPL monotherapy and further evaluated longitudinal scar improvement under this regimen.

## 2 Patients and Methods

### 2.1 Patients

Patients with early hypertrophic scars who underwent laser therapy were consecutively enrolled from February 2023 to May 2025 at the Aesthetic Outpatient Clinic of Nanjing Drum Tower Hospital. Written informed consent of all patients was obtained. Inclusion Criteria: 1. Diagnosed with hypertrophic scar. 2. Scar duration ≤ 6 months after formation. 3. Completion of the prescribed six treatment sessions. Exclusion Criteria: 1. History of oral/intradermal steroid therapy or other laser treatments prior to the study. 2. Systemic diseases affecting wound healing, such as diabetes, immune suppression, or coagulation dysfunction. 3. Known allergies to anesthetic agents or cooling gel used during the procedure. This study was approved by the Institutional Ethics Committee of Nanjing Drum Tower Hospital (Approval No. 2022–10‐901).

### 2.2 Study Design

**FIGURE 1:**
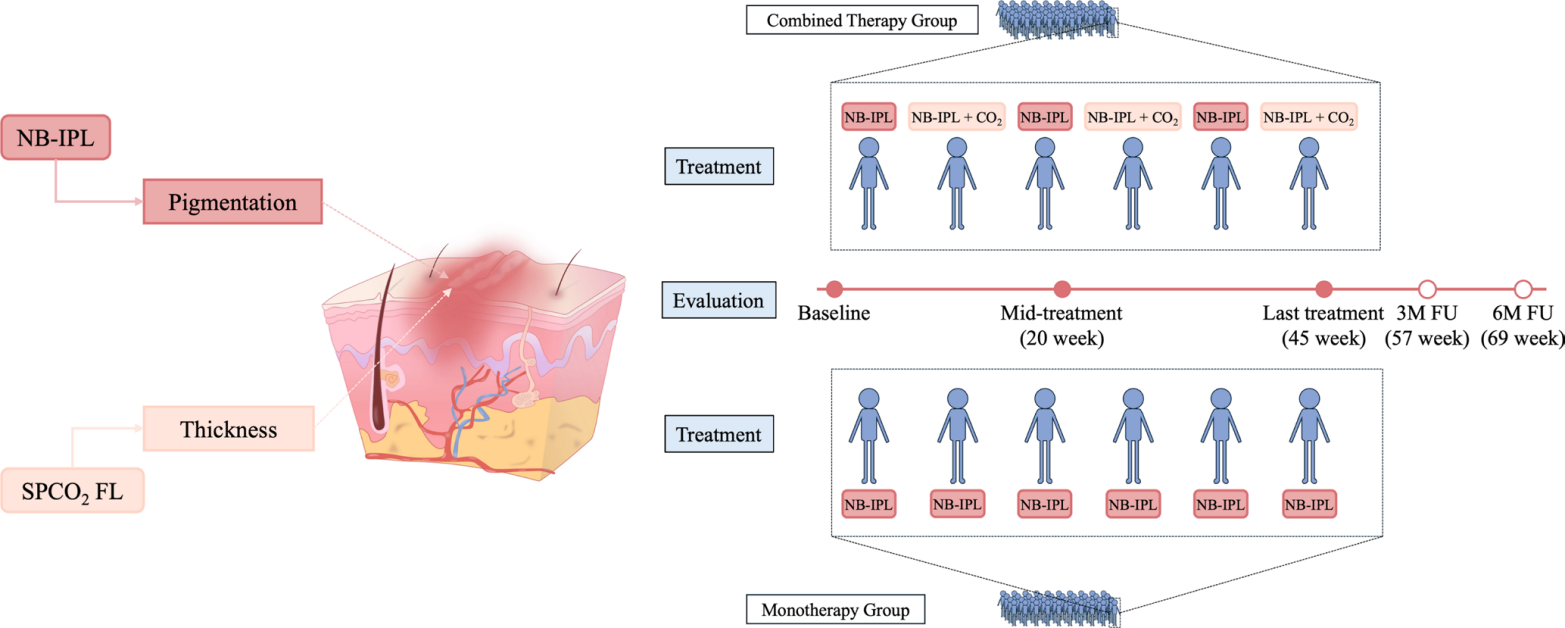
Treatment and Assessment Schedule. Patients with hypertrophic scars were retrospectively grouped into combined therapy or monotherapy. All patients completed six treatment sessions, with assessments at baseline, mid‐treatment, last treatment, and 3‐/6‐month (3 M/6 M) follow‐up (FU).

All patients underwent a standardized treatment regimen consisting of six sessions at intervals of 8–10 weeks. Clinical photographs were obtained at baseline, before and after each treatment session, and at the 3‐ and 6‐month post‐treatment follow‐up visits. All images were captured by the same photographer using a digital camera (D750, Nikon, Japan) under consistent positioning and lighting conditions. During the treatment of EBDs, standard safety protocols, including the use of specialized protective eyewear for both physicians and patients, were followed. A graphical abstract of the treatment and assessment schedule is provided in Figure 1.

### 2.3 Treatment Procedure

**FIGURE 2:**
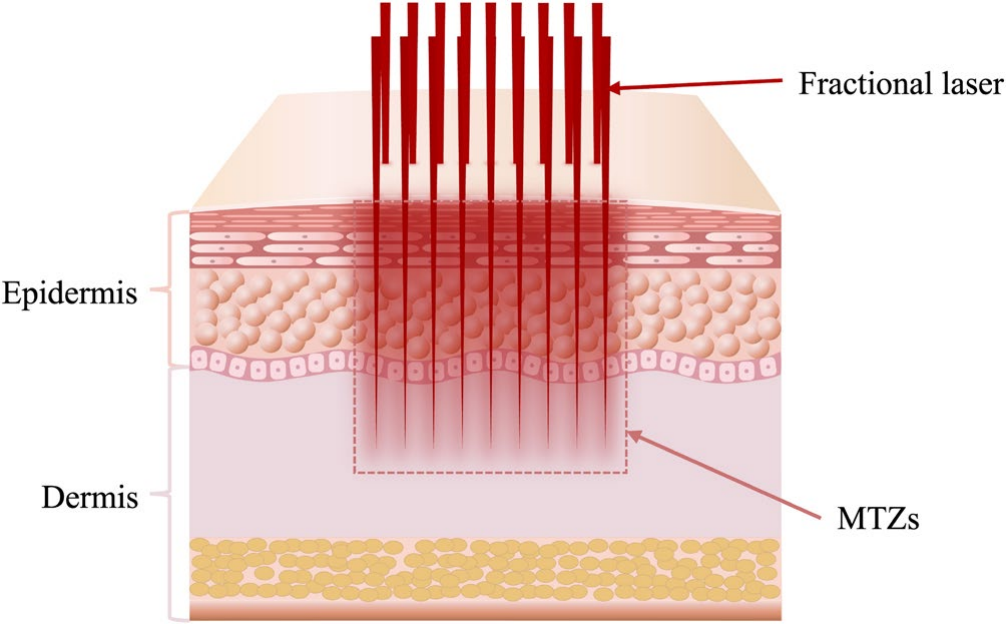
Schematic diagram of Microscopic Thermal Zones (MTZs).

For NB‐IPL monotherapy, ultrasound gel was evenly applied to the scar surface. The experienced operator used a NB‐IPL handpiece (Fotona Dynamis Pro, Fotona, Israel) with the following parameters: wavelength of 500–600 nm, spot size of 3 cm^2^, pulse width of 12–15 ms, and fluence of 7.5–10 J/cm^2^. Treatment started at a low energy setting on an inconspicuous perilesional region, with parameters adjusted gradually according to the patient's skin condition and individual tolerance. The clinical endpoint for each NB‐IPL session was defined as blurring of capillary vessels or mild darkening of the scar. For combination therapy, scars were topically pretreated with Compound Lidocaine Cream (Tsinghua Tongfang, China) under occlusion for 45 min. After complete cream removal, the treatment areas were disinfected with 70% alcohol. The NB‐IPL procedure was performed as described above. Subsequently, SPCO_2_ FL treatment was performed in DeepFX mode (Lumenis UltraPulse Encore CO_2_ Laser, Yokneam, Israel). Energy parameters were individualized according to scar severity: wavelength 10 600 nm, spot diameter 0.12 mm, density 5%–8%, frequency 200–300 Hz, and single‐pulse energy 20–50 mJ. Immediately after treatment, regular microscopic thermal zones (MTZs) formed on the treated skin, accompanied by visible scar contraction or flattening (Figure 2). All patients received immediate cold compresses for 20–30 min to minimize dermal thermal injury. Patients were instructed to avoid sun exposure and keep the treated area clean and dry post‐treatment. Topical silicone gel (Dermatix Ultra Gel, Menarini, Italy) was applied to promote epidermal repair.

### 2.4 Outcome Evaluation

Scar appearance was evaluated using the Vancouver Scar Scale (VSS), which was based on four parameters: pigmentation, vascularization, pliability, and thickness. The Patient and Observer Scar Assessment Scale (POSAS), a comprehensive tool consisting of two components: the Patient Scale (PS) (completed by the patient) and the Observer Scale (OS) (rated by two independent dermatologists) [9]. Specifically, the PS assesses pain, pruritus, color, pliability, thickness, and relief, while the OS evaluates vascularity, pigmentation, thickness, relief, pliability, and surface area. Furthermore, two subjective sensations, pain and pruritus, were analyzed using the data obtained from the patient‐reported scale. Treatment pain was evaluated using the Visual Analog Scale (VAS). VAS scores were documented during each treatment session and calculated as mean values. The VAS is a 0–10 linear scale, with higher scores indicating greater pain intensity.

### 2.5 Adverse Reactions

Postoperative adverse reactions, including prolonged pain, pruritus, local infection, blistering, scar depression, tissue atrophy, hyperpigmentation, hypopigmentation, and new scar formation, were recorded during each follow‐up visit.

### 2.6 Statistical Analysis

Normally distributed quantitative data are presented as mean ± standard deviation ($\bar{x}$ ± s), and intergroup comparisons are performed using two independent sample *t*‐tests. Count data are expressed as examples or percentages [*n* (%)], and Pearson's chi‐square test is used for intergroup comparisons. For longitudinal data, repeated‐measures ANOVA (RM‐ANOVA) is employed; for comparing simple effects between groups, two‐factor RM‐ANOVA is used; and for comparing simple effects within groups, single‐factor RM‐ANOVA and paired sample *t*‐tests are applied [10]. A *p*‐value of less than 0.05 is considered statistically significant. All data were analyzed using SPSS version 26.0.

## 3 Results

### 3.1 Patient Information

Among 100 initially assessed patients, 14 were excluded due to incomplete clinical data or loss to follow‐up, and 86 were eligible and included in the final analysis. The overall mean age of the participants was 28.90 ± 8.37 years, with a mean scar duration of 2.21 ± 0.88 months. Baseline characteristics were comparable between the NB‐IPL + CO_2_ and NB‐IPL groups, with no significant differences in gender distribution, age, scar duration, VAS scores, or scar location (Table 1). No severe postoperative complications were identified from retrospective review of medical records in all 86 patients.

**TABLE 1:** Baseline characteristics and VAS pain scores.

Characteristics	NB‐IPL + CO_2_	NB‐IPL	*x* ^2^	*t*	*p*
Gender (M vs. F)	17:29	17:23	0.275	—	0.600
Age (years)	28.98 ± 8.62	28.43 ± 8.89	—	0.293	0.771
Scar duration (months)	2.11 ± 0.87	2.33 ± 0.88	—	−1.135	0.259
VAS	4.04 ± 1.51	4.25 ± 1.43	—	−0.650	0.517
Scar Location					
Head and Neck	30	29	0.530	—	0.767
Extremities	10	7			
Trunk	6	4			

### 3.2 Patient and Observer Scar Assessment Scale

#### 3.2.1 Patient Scale

Both groups showed significant reductions in POSAS Patient Scale (PS) scores over time, and the NB‐IPL + CO_2_ group demonstrated a significantly greater improvement than the NB‐IPL group. Specifically, while baseline scores were comparable (*p* = 0.779), the NB‐IPL + CO_2_ group achieved significantly lower PS scores than the NB‐IPL group starting after 3 treatment sessions (approx. 16–20 weeks; *p* = 0.035). This difference became more pronounced after 6 treatment sessions and persisted through the 6‐month follow‐up. Detailed statistical comparisons are presented in Table 2.

**TABLE 2:** Comparison of patient scale scores over time.

Group	Baseline	Mid‐treatment	Last treatment	3‐month FU	6‐month FU	*F*	*p*
NB‐IPL + CO_2_	26.02 ± 5.08	18.33 ± 4.64^a^	12.39 ± 4.04^ab^	9.96 ± 3.02^abc^	8.24 ± 1.70^abcd^	707.146	0.001*
NB‐IPL	26.38 ± 6.53	20.75 ± 5.86^a^	16.50 ± 5.41^ab^	14.28 ± 4.68^abc^	12.26 ± 3.63^abcd^	535.934	0.001*
*t*	−0.282	−2.140	−4.025	−5.002	−6.341		
*p*	0.779	0.035*	0.001*	0.001*	0.001*		
*F*_Group_ = 10.896; *F*_Time_ = 1203.175; *F*_Int_. = 19.422							

*Note:* ^a^versus baseline, ^b^versus mid‐treatment, ^c^versus last treatment, ^d^versus 3‐month FU, *p* < 0.05 for all marked comparisons; *, significant difference.

Abbreviations: *F*, F‐statistic; FU, follow‐up; *p*, *p*‐value.

Patient‐reported pain and pruritus scores are shown in Table 3. Both the combined (D + C) and monotherapy (D) groups demonstrated significant reductions in pain and pruritus over time. For pain, repeated‐measures ANOVA revealed significant group, time, and interaction effects, indicating greater and faster pain relief in the combined group. For pruritus, while the time effect was significant, no significant between‐group or interaction differences were observed.

**TABLE 3:** Pain and pruritus scores over time.

	Baseline	Mid‐treatment	Last treatment	3‐month FU	6‐month FU	*F*	*p*	Group		Time		Int.	
								*F*	*p*	*F*	*p*	*F*	*p*
Pain													
D + C	2.72 ± 0.81	1.72 ± 0.58^a^	1.20 ± 0.40^ab^	1.15 ± 0.36^ab^	1.02 ± 0.15^abcd^	135.868	0.001*	7.352	0.008*	269.873	0.001*	2.849	0.024*
D	2.92 ± 0.74	2.18 ± 0.82^a^	1.56 ± 0.64^ab^	1.33 ± 0.53^abc^	1.13 ± 0.34^abcd^	139.115	0.001*						
Pruritus													
D + C	2.76 ± 0.87	1.85 ± 0.70^a^	1.30 ± 0.51^ab^	1.17 ± 0.44^abc^	1.02 ± 0.15^abcd^	130.271	0.001*	3.853	0.053	250.098	0.001*	1.755	0.172
D	2.98 ± 1.00	2.25 ± 0.93^a^	1.60 ± 0.74^ab^	1.35 ± 0.58^abc^	1.10 ± 0.30^abcd^	121.325	0.001*						

*Note:* ^a^versus baseline, ^b^versus mid‐treatment, ^c^versus last treatment, ^d^versus 3‐month FU, *p* < 0.05 for all marked comparisons; *, significant difference.

Abbreviations: D, NB‐IPL monotherapy; D + C, combined NB‐IPL and SPCO_2_ FL; *F*, F‐statistic; FU, follow‐up; *p*, *p*‐value.

#### 3.2.2 Observer Scale

POSAS Observer Scale (OS) scores decreased significantly over time in both groups, with the NB‐IPL + CO_2_ group showing significantly better outcomes. In detail, the OS scores showed statistically significant differences between the two groups emerging at 16–20 weeks (*p* = 0.034) and widening progressively until the 6‐month follow‐up (Table 4).

**TABLE 4:** Comparison of observer scale scores over time.

Group	Baseline	Mid‐treatment	Last treatment	3‐month FU	6‐month FU	*F*	*p*
NB‐IPL + CO_2_	26.74 ± 5.98	20.07 ± 5.37^a^	13.93 ± 4.92^ab^	9.70 ± 3.46^abc^	7.96 ± 2.17^abcd^	716.047	0.001*
NB‐IPL	27.30 ± 6.72	22.70 ± 5.97^a^	18.20 ± 5.68^ab^	16.12 ± 5.27^abc^	13.25 ± 4.40^abcd^	818.960	0.001*
*t*	−0.409	−2.155	−3.734	−6.584	−6.919		
*p*	0.683	0.034*	0.001*	0.001*	0.001*		
*F*_Group_ = 13.146; *F*_Time_ = 1365.007; *F*_Int_. = 41.034							

*Note:* ^a^versus baseline, ^b^versus mid‐treatment, ^c^versus last treatment, ^d^versus 3‐month FU, *p* < 0.05 for all marked comparisons; *, significant difference.

Abbreviations: *F*, F‐statistic; FU, follow‐up; *p*, *p*‐value.

### 3.3 The Comparison of Individual VSS Parameters

**FIGURE 3:**
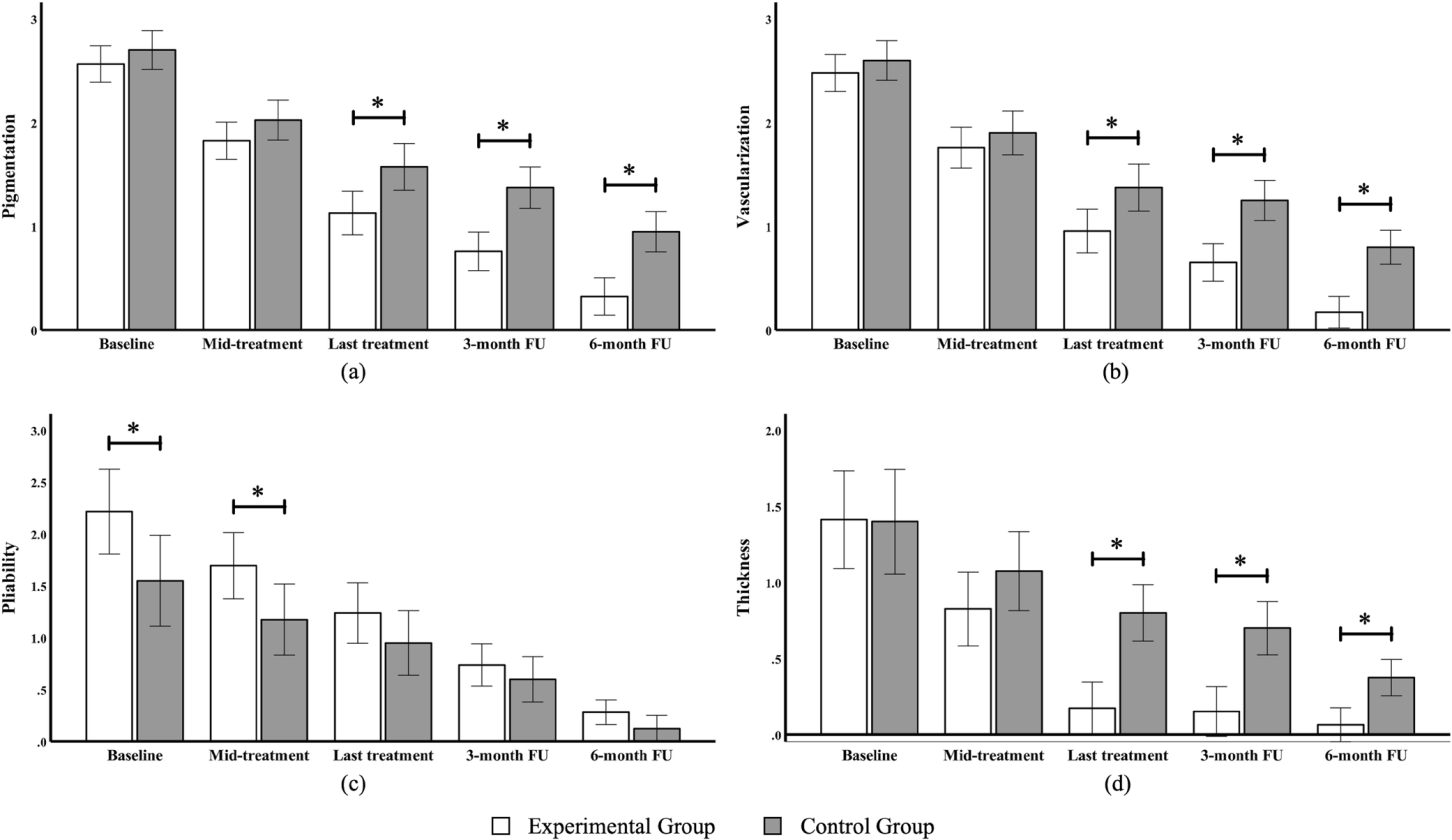
Individual VSS parameters over time for the experimental and control groups. Data are presented as mean ± SD. FU, Follow‐up. Asterisks (*) indicate statistically significant differences between the two groups.

The single‐factor RM‐ANOVA results indicated a significant time effect for all VSS parameters, demonstrating that both treatment protocols effectively improved scar conditions over time (Table 5). Regarding the group effect, significant differences were observed across all parameters, with the combined (D + C) group showing greater overall improvement compared to the monotherapy (D) group. Furthermore, significant interaction effects (group × time) were found for all parameters, suggesting that the improvement trends for pigmentation, vascularization, pliability, and thickness differed significantly between the two groups, with the combined group exhibiting a more rapid or pronounced reduction in scores, particularly after 6 treatment sessions. The specific inter‐group differences at each time point are visualized in Figure 3.

**TABLE 5:** Comparison of individual VSS parameters over time.

Parameters	Baseline	Mid‐treatment	Last treatment	3‐month FU	6‐month FU	*F*	*p*	Group		Time		Int.	
								*F*	*p*	*F*	*p*	*F*	*p*
Pigmentation													
D + C	2.57 ± 0.58	1.83 ± 0.64^a^	1.13 ± 0.78^ab^	0.76 ± 0.64^abc^	0.33 ± 0.47^abcd^	197.054	0.001*	12.947	0.001*	303.374	0.001*	6.561	0.001*
D	2.70 ± 0.61	2.03 ± 0.58^a^	1.58 ± 0.64^ab^	1.37 ± 0.63^abc^	0.95 ± 0.75^abcd^	115.385	0.001*						
Vascularization													
D + C	2.48 ± 0.59	1.76 ± 0.71^a^	0.96 ± 0.76^ab^	0.65 ± 0.57^abc^	0.17 ± 0.38^abcd^	185.962	0.001*	12.152	0.001*	307.596	0.001*	7.000	0.001*
D	2.60 ± 0.63	1.90 ± 0.63^a^	1.38 ± 0.67^ab^	1.25 ± 0.67^abc^	0.80 ± 0.65^abcd^	130.595	0.001*						
Pliability													
D + C	2.22 ± 1.37	1.70 ± 1.09^a^	1.24 ± 0.95^ab^	0.74 ± 0.68^abc^	0.28 ± 0.46^abcd^	63.129	0.001*	4.068	0.047*	100.825	0.001*	3.176	0.045*
D	1.55 ± 1.43	1.17 ± 1.08^a^	0.95 ± 1.04^ab^	0.60 ± 0.71^abc^	0.13 ± 0.34^abcd^	41.018	0.001*						
Thickness													
D + C	1.41 ± 1.02	0.83 ± 0.64^a^	0.17 ± 0.38^ab^	0.15 ± 0.36^ab^	0.07 ± 0.25^abcd^	84.955	0.001*	6.373	0.013*	111.395	0.001*	7.898	0.001*
D	1.40 ± 1.17	1.07 ± 1.00^a^	0.80 ± 0.76^ab^	0.70 ± 0.72^abc^	0.38 ± 0.49^abcd^	36.202	0.001*						

*Note:* ^a^versus baseline, ^b^versus mid‐treatment, ^c^versus last treatment, ^d^versus 3‐month FU, *p* < 0.05 for all marked comparisons; *, significant difference.

Abbreviations: D, NB‐IPL monotherapy; D + C, combined NB‐IPL and SPCO_2_ FL; *F*, F‐statistic; FU, follow‐up; *p*, *p*‐value.

### 3.4 Typical Cases

**FIGURE 4:**
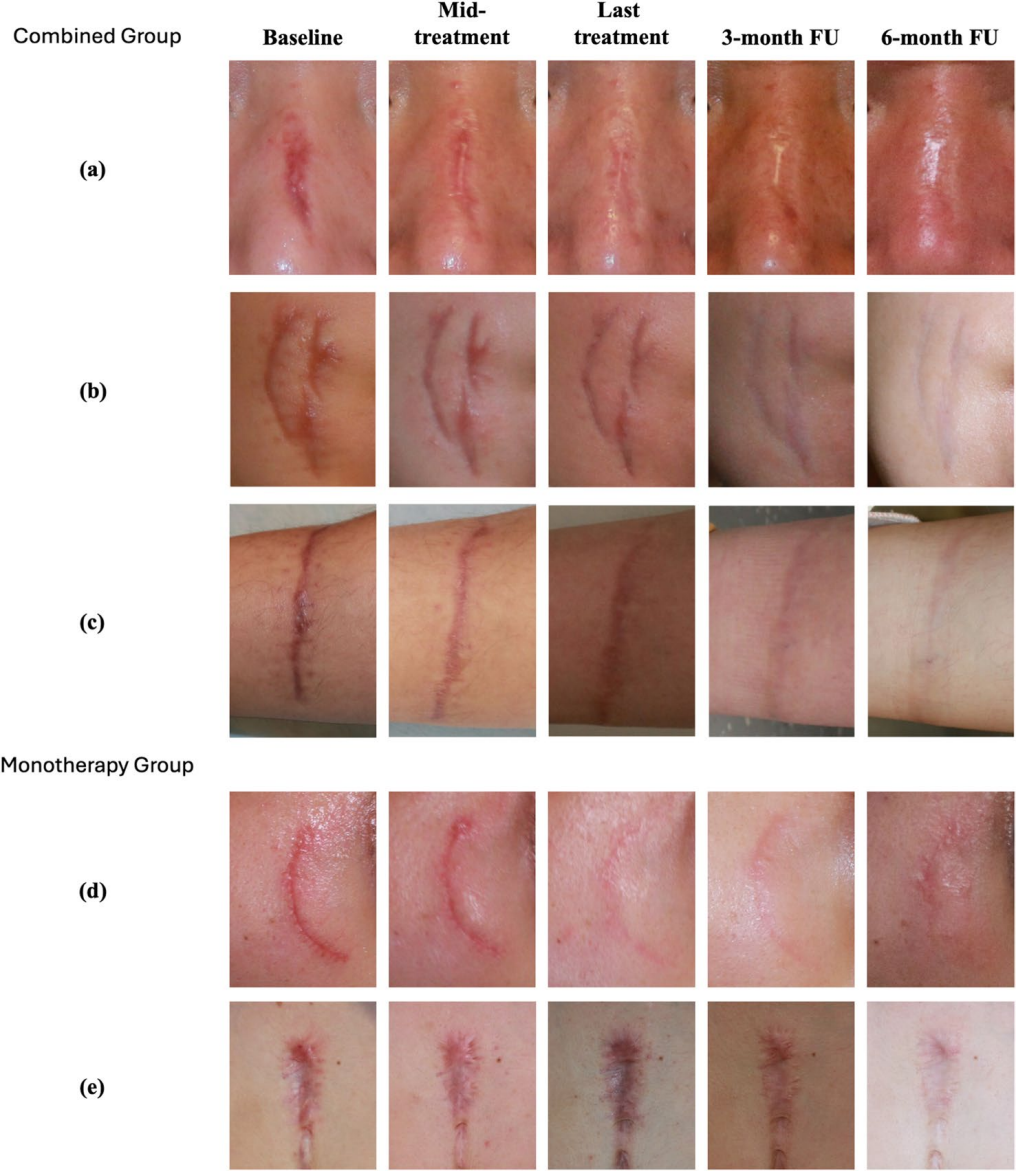
Cases in the combined group (a–c) and the monotherapy group (d, e) at baseline, during treatment, and at two follow‐up (FU) time points. Combined Group: (a) A 38‐year‐old male with HTS on the nasal bridge. (b) A 26‐year‐old male with HTS on the right perioral region. (c) A 16‐year‐old female with HTS on the medial right lower leg. Monotherapy Group: (d) A 31‐year‐old male with HTS on the left infraorbital region. (e) A 23‐year‐old female with HTS on the mid‐chest.

Representative clinical cases are shown in Figure 4. Compared with NB‐IPL monotherapy, the combined NB‐IPL + SPCO_2_ FL regimen yielded greater improvements in scar pigmentation, thickness, stiffness, pliability, and vasculature via the photothermolysis mechanism.

## 4 Discussion

Although the pathological mechanism of HTS formation has not been clearly elucidated, it is widely accepted that HTS represents a chronic inflammatory state and an excessive vascular response to tissue trauma [11]. Following skin injury, numerous inflammatory mediators are released to drive the differentiation of fibroblasts into myofibroblasts, which promote scar thickening and contraction through the secretion and expression of α‐smooth muscle actin (α‐SMA) [12]. Meanwhile, excessive microvascular proliferation creates a permissive microenvironment that maintains fibroblast viability and collagen secretion via the delivery of oxygen, nutrients, and signaling molecules across endothelial channels [13]. Furthermore, insufficient collagenase activity in HTS leads to impaired degradation of accumulated collagen, as vessel‐targeted monotherapies fail to effectively reduce preformed collagen deposits. Therefore, inhibiting collagen synthesis by fibroblasts is more critical than removing preformed collagen, but clearance of existing collagen remains necessary in HTS management [14].

As early intervention techniques for HTS, EBDs could induce thermal coagulation of microvessels and ablate collagen within the dermis. Their core mechanism relies on selective photothermolysis: light energy absorbed by endogenous chromophores is converted into thermal or mechanical energy or mediates photobiomodulation, ultimately disrupting or regulating target tissues [15]. Peripheral low‐thermal zones also trigger heat shock responses that promote tissue repair and regeneration [16]. With short pulse durations, rapid thermal relaxation of target chromophores and built‐in cooling systems, EBDs can fully treat the scars before heat spreads to surrounding tissues, effectively limiting thermal damage and enhancing clinical safety [17]. Common chromophores targeted by these modalities are hemoglobin, melanin, and water. Specifically, NB‐IPL acts on hemoglobin to reduce erythema, whereas SPCO_2_ FL targets water for epidermal vaporization.

Intense pulsed light (IPL) has long been used for HTS treatment and is commonly defined as a vascular‐targeted device [18]. By targeting hemoglobin in intravascular erythrocytes, IPL (400–1200 nm) occludes local blood vessels and reduces the blood supplement required for scar tissue growth. Histopathological studies have demonstrated that IPL therapy upregulates the proliferation viability of fibroblasts and vascular endothelial cells [19]. However, IPL exerts no obvious effects on the secretion levels of vascular endothelial growth factor and matrix metalloproteinases (MMPs), nor does it alter cell morphology. MMPs are known to specifically degrade most components of the extracellular matrix (ECM). Derived from IPL, NB‐IPL delivers higher precision. It targets the absorption spectrum of oxyhemoglobin by utilizing a filter to capture light within the 500–600 nm wavelength range. Moreover, replacing attenuated waves with rectangular waves enhances single‐pulse stability, thereby improving treatment safety and efficacy [20].

Carbon dioxide laser has been utilized for the management of HTS since the mid‐1980s. Emitting coherent light at a wavelength of 10 600 nm, it selectively targets water molecules within scar tissue [16]. Thermal energy generated during tissue vaporization disrupts irregular collagen fibers and attenuates excessive collagen deposition, ultimately leading to scar softening and flattening. The fractional technique divides the extensive damage into tiny microspots separated by undamaged tissue, which enhances safety and accelerates skin re‐epithelialization [21]. Meanwhile, this laser penetrates the dermal to a depth of 400–1000 μm, effectively remodeling disorganized collagen fibers. SPCO_2_ FL facilitates cutaneous repair and reduces the thickness and density of collagen bundles by upregulating the expression of heat shock protein 70, basic fibroblast growth factor, elastin, and MMPs, while downregulating mRNA levels of transforming growth factor‐β, α‐SMA, and type I/III procollagen [22, 23, 24]. However, some studies have yielded inconsistent or negative outcomes, reporting no meaningful changes in the type I/III procollagen mRNA ratio after SPCO_2_ FL treatment [25, 26].

It is widely accepted that the combination therapy with EBDs yields better skin repair outcomes than monotherapy, and the combination of IPL and CO_2_ laser constitutes the most effective therapeutic strategy [27]. Daoud et al. reported that combined therapy with IPL and CO_2_ ablative fractional laser (AFL) yielded greater average clinical improvements versus no treatment and monotherapy [28]. Nevertheless, their study was limited by insufficient sample size and the experimental design wherein CO_2_ AFL was used as the primary therapeutic modality. Zhang et al. further validated that combination therapy with IPL plus CO_2_ AFL induced significantly better POSAS score improvements and a higher patient satisfaction rate (100% vs. 84%) [29]. However, their work was constrained by insufficient follow‐up visits and short follow‐up duration. To address existing limitations, we retrospectively analyzed clinical data from 86 patients with early HTS. All patients were allocated into two groups and received six treatment sessions at intervals of 8–10 weeks, with longitudinal clinical assessment performed at five time points. The statistical outcomes indicated that the combined group achieved superior reductions in POSAS and VSS scores, accompanied by marked alleviation of pain and pruritus.

Further analysis of the data revealed a significant difference in pain and pruritus symptoms between the two groups during treatment periods but no difference after treatment. This difference may be due to the stabilization of symptom severity following treatment and the excellent efficacy of early intervention. In addition, intergroup comparisons revealed significant differences in pruritus regarding time effects, while no group and interaction effects were observed. Previous studies have demonstrated that scar pruritus is closely linked to hyperemia and the release of pruritogenic mediators (e.g., histamine and substance P) from mast cells and endothelial cells [30, 31]. As the main intervention in this study, NB‐IPL exerts selective photothermolysis on dermal microvessels, which may reduce local inflammation and the release of itch‐inducing substances and might in turn alleviate pruritus [32]. However, further studies are warranted to fully elucidate the underlying mechanisms.

This study has several limitations. First, the retrospective single‐center design may introduce selection bias, which can be alleviated by future multicenter prospective cohort studies. Second, the modest sample size limits extrapolation of our findings, and enrollment of larger sample cohorts is essential to strengthen result generalizability. Third, present evaluations mainly depend on subjective scales, while objective assessment tools such as histopathological examination or computer‐assisted image analysis would facilitate further validation of the results. Despite these inherent limitations, uniform treatment regimens and longitudinal efficacy assessments may support the external validity of our findings in similar clinical settings.

## 5 Conclusions

In summary, this study demonstrated that combined treatment with NB‐IPL and SPCO_2_ FL achieved significantly superior clinical efficacy for hypertrophic scars compared with NB‐IPL monotherapy, as evidenced by improved scar appearance, pliability, and patient symptoms. However, considering the retrospective design, relatively small sample size, and subjective evaluation, the findings should be interpreted cautiously. Further prospective, large‐sample studies are needed to confirm these results.

## Author Contributions

Conception and design: Yanan Jiang and Yadan Chen; Administrative support: Qian Tan and Yanan Jiang; Provision of study materials or patients: Yanan Jiang, Yadan Chen, and Qian Tan; Collection and assembly of data: Yadan Chen, Yanan Jiang, Demei Zhao; Data analysis and interpretation: all authors; Manuscript writing: all authors; Final approval of manuscript: all authors.

## Ethics Statement

Ethical approval for the study was granted by the Institutional Ethics Committee of Nanjing Drum Tower Hospital (approval No. 2022‐10‐901). All patients had previously provided written informed consent for the use of their clinical data.

## Conflicts of Interest

The authors declare no conflicts of interest.

## Data Availability

The data that support the findings of this study are available from the corresponding author upon reasonable request.
